# PFO Closure after Pulmonary Valve Intervention in Patients with Hypoxemia Due to Right-to-Left Intracardiac Shunt

**DOI:** 10.14797/mdcvj.1153

**Published:** 2022-10-03

**Authors:** Suhas Babu, Zhihao Zhu, Miguel Chavez, Rahul Singh, Zhongyu Li, Lamees I. El Nihum, Valeria E. Duarte, Thomas E. MacGillivray, C. Huie Lin

**Affiliations:** 1 Methodist DeBakey Heart & Vascular Center, Houston Methodist Hospital, Houston, Texas, US; 2 Texas A&M University, College Station, Texas, US; 3 University of Utah, Salt Lake City, Utah, US; 4 University of Minnesota, Minneapolis, Minnesota, US; 5 Baylor College of Medicine, Houston, Texas, US

**Keywords:** patent foramen ovale, hypoxemia, right-to-left intracardiac shunt, right ventricle, volume overload, pulmonary valve regurgitation, pulmonary valve replacement

## Abstract

Four patients with pulmonary valve (PV) disease and patent foramen ovale (PFO) presented with dyspnea on exertion. Work-up revealed hypoxemia secondary to right-to-left intracardiac shunt. We demonstrate that correction of the primary culprit right heart overload lesion via PV replacement enabled safe PFO repair and resolution of hypoxemia.

## Introduction

Early surgical or percutaneous intervention for pulmonary valve (PV) disease may lead to later development of restenosis or regurgitation. Long-standing PV pathology can cause right ventricular (RV) volume overload with subsequent right heart dysfunction. In the setting of RV volume and pressure overload due to PV disease, patent foramen ovale (PFO) may have a beneficial role in allowing decompression of the right heart and enabling left side forward output but with the opportunity cost of hypoxia. In addition, abrupt PFO closure could lead to right heart decompensation. Here, we propose that correction of the primary culprit right heart overload lesion may enable safe PFO repair. We present four patients with PFO and hypoxemia secondary to right-to-left shunt in the setting of RV volume overload due to PV regurgitation. All of them underwent successful PFO closure and resolution of hypoxia after PV replacement.

## Case 1

A 47-year-old male with a history of tetralogy of Fallot repaired at childhood, patch augmentation of pulmonary artery (PA) bifurcation, and RV-PA homograft at age 30 presented with dyspnea on exertion. He underwent cardiac magnetic resonance (CMR) and cardiac catheterization, which demonstrated right-to-left shunt and PV (Video 1) and tricuspid valve (TV) regurgitation (Table 1). Cardiac catheterization oximetry revealed arterial oxygen saturation of 88% to 92%. Given continued symptoms of dyspnea, near syncope, and advancing RV dysfunction, he was taken to the catheterization lab for PV intervention with balloon dilation, PALMAZ™ XL P4010 stent (Cordis) and Melody™ valve (Medtronic) placement (Video 2). Subsequently, oxygen saturation improved dramatically. Intracardiac echocardiography demonstrated reduced but persistent right-to-left shunting (Video 3); thus, the defect was closed with a 25 mm Amplatzer PFO occluder (Abbott Laboratories) (Video 4). At 3-month follow-up, the patient’s exercise tolerance and ambulatory saturation had normalized.

**Table 1:** Hemodynamic parameters of cardiac catheterization and cardiac magnetic resonance imaging. PA: pulmonary artery; PCWP: pulmonary capillary wedge pressure; PVR: pulmonary valve regurgitation; RA: right atrium; RV: right ventricle; RVEDVi: right ventricular end-diastolic volume index; TVR: tricuspid valve regurgitation.


	CARDIAC CATHETERIZATION						CARDIAC MAGNETIC RESONANCE IMAGING		
	
	RA (mm Hg)	RV (mm Hg)	PA (mm Hg)	PVR (Wu)	PCWP (mm Hg)	Shunt Fraction (Qp/Qs)	RVEDVi (mL)	TVRSeverity	PVR Severity

Case 1	19	44/23	30/20	1.3	18	0.9	152	Mild	Moderate to severe

Case 2	12	45/10	45/12	2	16	0.93	165	Moderate	Severe

Case 3	20	72/20	60/30	2.5	22	0.77	207	Severe	Severe

Case 4	13	27/9	28/10	1	14	0.69	143	Moderate	Severe


**Video 1:** Main pulmonary angiography demonstrating degenerated right ventricle-pulmonary artery homograft with pulmonic regurgitation; also at https://youtube.com/shorts/10_v97YnH3Q.

**Video 2:** Main pulmonary angiography demonstrating prosthetic pulmonic valve competence following implantation of Palmaz XL P4010 stent and Melody valve; also at https://youtube.com/shorts/Fnbif5eio0s.

**Video 3:** Intracardiac echocardiography performed from the right atrium with color Doppler demonstrating right to left shunt through the PFO at baseline; also at https://youtu.be/OOFEhr55Sgg.

**Video 4:** Intracardiac echocardiography demonstrating no residual right to left shunt through the PFO following implantation of a 25mm Amplatzer PFO Occluder (Abbott, Santa Clara); also at https://youtu.be/xBEzK-gxYK4.

## Case 2

**Figure 1:**
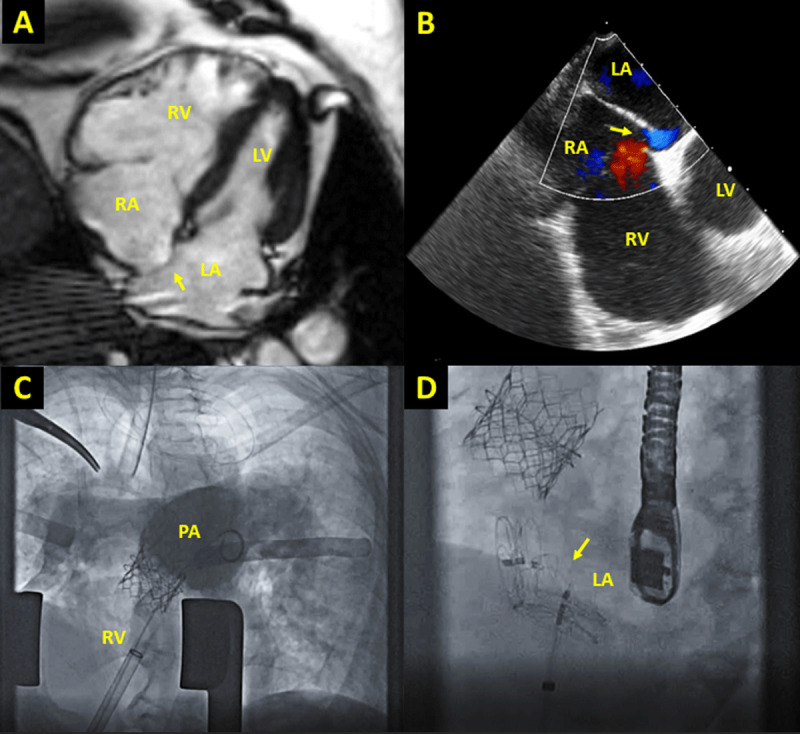
Case imaging. **(A)** Four-chamber cardiac magnetic resonance imaging demonstrating severely dilated RV compared to LV with leftward bowing of interatrial septum (arrow). **(B)** Transesophageal echocardiography demonstrating right-to-left shunt through PFO (arrow). **(C)** Fluoroscopic images from hybrid pulmonary valve replacement and PFO closure; angiogram demonstrates no residual pulmonic regurgitation. **(D)** Two occluders (arrow) were deployed to close the PFO and atrial septal deflect. LA: left atrium; LV: left ventricle; PA: pulmonary artery; PFO: patent foramen ovale; RA: right atrium; RV: right ventricle.

A 67-year-old female with chronic obstructive pulmonary disease and congenital pulmonary stenosis who underwent surgical valvotomy at age 8 presented with progressive dyspnea on exertion. She was hospitalized shortly before encounter for acute heart failure exacerbation, requiring aggressive diuresis. CMR and cardiac catheterization revealed right-to-left shunt and PV and TV regurgitation (Table 1, Figure 1 A, B). On 6-minute walk test, her arterial oxygen saturation decreased to 87%. Pulmonary function tests revealed obstructive pattern with forced expiratory volume of 40%. As her prior surgical transannular patch yielded a PV annulus too large for transcatheter PV, we proceeded with hybrid intervention to avoid cardiopulmonary bypass. She underwent PA banding and per-ventricular balloon-expandable PV replacement with a 29-mm Edwards SAPIEN 3 valve (Edwards Lifesciences) as well as closure of a fenestrated atrial septal defect (ASD) and PFO with two 25-mm CARDIOFORM Septal Occluders (WL Gore & Associates, Inc) (Figure 1 C, D). Angiography confirmed excellent competency of the PV, and transesophageal echocardiography showed no residual shunt through the PFO. At 4-month follow-up, she had improved exercise tolerance and no hypoxia at rest.

## Case 3

**Figure 2:**
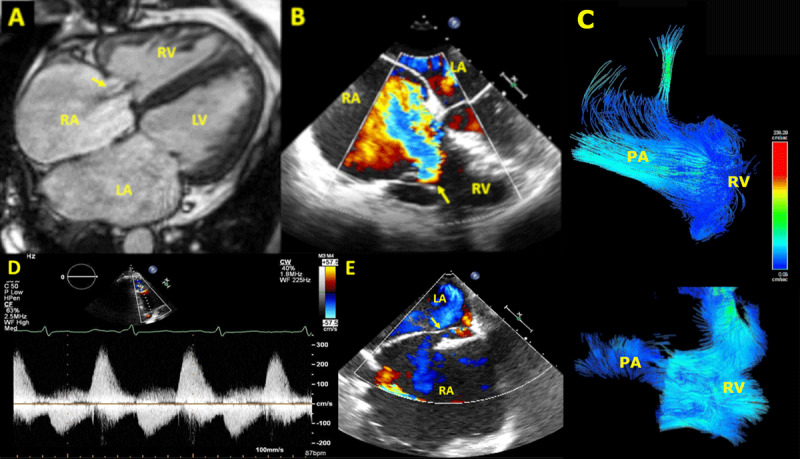
Severe PV regurgitation leading to severe functional TV regurgitation, favoring a right-to-left shunt via PFO. Severe TV regurgitation jet shown by 4-chamber view **(A)** CMR and **(B)** transesophageal echocardiography. **(C)** Four-dimensional CMR demonstrating severe PV regurgitation. **(D)** Spectral doppler showing classic PV regurgitation waveform. **(E)** PFO with right-to-left shunt by transesophageal echocardiography. LA: left atrium; LV: left ventricle; PA: pulmonary artery; PFO: patent foramen ovale; PV: pulmonary valve; RA: right atrium; RV: right ventricle; TV: tricuspid valve.

A 49-year-old female with congenital pulmonic stenosis who underwent valvotomy at age 2 presented with dyspnea and cyanosis on exertion. CMR and cardiac catheterization demonstrated right-to-left shunt and PV and TV regurgitation (Table 1, Figure 2). She underwent surgical PV replacement, TV annuloplasty, and primary PFO closure. At 2-month follow-up, exercise tolerance improved with oxygen saturation of 95% to 98% on room air. Transthoracic echocardiography at 7 months showed reduced RV enlargement with RV end-diastolic volume index of 87 mL/m^2^.

## Case 4

A 63-year-old male with a history of tetralogy of Fallot repaired in adulthood presented with worsening dyspnea on exertion and recurrent cyanosis for 2 years. CMR and cardiac catheterization revealed right-to-left shunt and PV and TV regurgitation (Table 1). He subsequently underwent PV replacement with a 27-mm Avalus valve (Medtronic), RV outflow tract reconstruction with bovine pericardium, and closure of his PFO (Figure 1 D–F). At 4-month follow-up, the patient denied cyanosis and reported improved exercise tolerance.

## Discussion

We have presented four cases of right heart volume overload due to PV regurgitation that led to increased RV strain and subsequently translated to increased right atrial pressures, ultimately inducing increased right-to-left shunt through the PFO as a maladaptive response to decompress the right heart. Physical exertion further increased right-to-left shunting manifesting as worsening hypoxia and cyanosis. We discussed proceeding with PFO closure to reduce exertional hypoxia; however, to avoid acute increase in RV load and the risk for RV failure, we pursued PV replacement first. Following resolution of the primary hemodynamic lesion, we hypothesized that hemodynamic adaptation was no longer necessary, and PFO closure could be performed safely.

PFO has been associated with exacerbating hypoxemic medical conditions manifesting as dyspnea and sometimes as platypnea-orthodeoxia syndrome in the absence of pulmonary hypertension.^1,2^ This is presumed to be secondary to an increase in right-to-left shunting once right-sided pressures overcome the higher left-sided pressures, which keep the septum primum pressed against the septum secundum.^3^ Currently, there are limited randomized clinical trials (eg, PCOSA 1, DIVER-PFO) that evaluate the effect of PFO closure and correction of hypoxemia in hypoxemic medical conditions; however, these studies have not yet shown any concluding evidence that favors benefit of closing PFO in hypoxemic medical conditions.

Like these clinical scenarios, there have been few cases reported in which a PFO may have functioned as a means of decompression of a high-pressure system. A case report by Giuseppe et al. reported a patient with hypertrophic cardiomyopathy with significant left-to-right shunting through a PFO.^4^ Hemodynamic evaluation revealed a significant increase in left ventricular end-diastolic pressure when occluding the PFO.^4^

Very commonly, as in our case series, right-to-left shunting through PFO occurs due to volume overload of the RV, in which the RV dilates to compensate for higher volume load. This volume overload is known to be caused by conditions such as TV and PV regurgitation along with ASD, conditions that have been widely studied.^5^ Currently, closure of PFO in RV volume overload is not routinely performed, and evidence so far has only shown promising effects in limited circumstances and remains largely subject to a case-by-case basis. Careful hemodynamic and symptomatic assessment should be made before attempting closure. In this series, we have presented scenarios wherein PFO closure and resolution of hypoxemia were successfully performed after treatment of the underlying RV volume overload lesion. Quality of life was markedly improved in these patients after PFO closure when combined with reduction of RV volume overload due to pulmonic regurgitation via PV replacement. Larger studies examining quantitative hemodynamic, anatomic, and physiologic data to enable safe PFO closure will help guide clinicians on which patients will tolerate PFO closure in right heart pathology.

## Conclusion

We have presented a series of cases in which PFO may have played a hemodynamically advantageous role in right heart decompression, with subsequent successful PFO closure following PV replacement. We propose that correction of the primary culprit right heart overload lesion may enable safe PFO repair once decompression is no longer necessary.

## Key Points

- Right-to-left shunting through patent foramen ovale (PFO) results from volume overload of the right ventricle, which dilates to compensate for higher volume load.
- PFO may play an advantageous role in right heart decompression, with successful PFO closure and resolution of hypoxemia following correction of the primary culprit right heart overload lesion.
- Further studies are needed to determine the individualized need for PFO closure based on the specific hemodynamic, anatomic, and physiologic parameters of the patient.
